# Supplementary material for: Manganese Oxide Nanoparticles as Safer Seed Priming Agent to Improve Chlorophyll and Antioxidant Profiles in Watermelon Seedlings
Source: Nanomaterials (Basel). 2021 Apr 15;11(4):1016. doi: 10.3390/nano11041016 (PMC8071577; doi:10.3390/nano11041016)
Supplement: Supplementary file 1 [file nanomaterials-11-01016-s001.zip › nanomaterials-1138921-supplementary.pdf]

nanomaterials

Supplementary Materials

Manganese Oxide Nanoparticles as Safer Seed Priming Agent to Improve Chlorophyll and Antioxidant Profiles in Watermelon Seedlings

Deepak M. Kasote, Jisun H.J. Lee, Guddarangavvanahally K. Jayaprakasha and Bhimanagouda S. Patil *

Vegetable and Fruit Improvement Center, Department of Horticultural Sciences, Texas A&M University, 1500 Research Parkway, Suite A120, College Station, TX 77845-2119, USA; deepakkasote06@gmail.com (D.M.K.); jslee@tamu.edu (J.H.J.L.); gkjp@tamu.edu (G.K.J.)

* Correspondence: b-patil@tamu.edu; Tel.: +1-979-458-8090

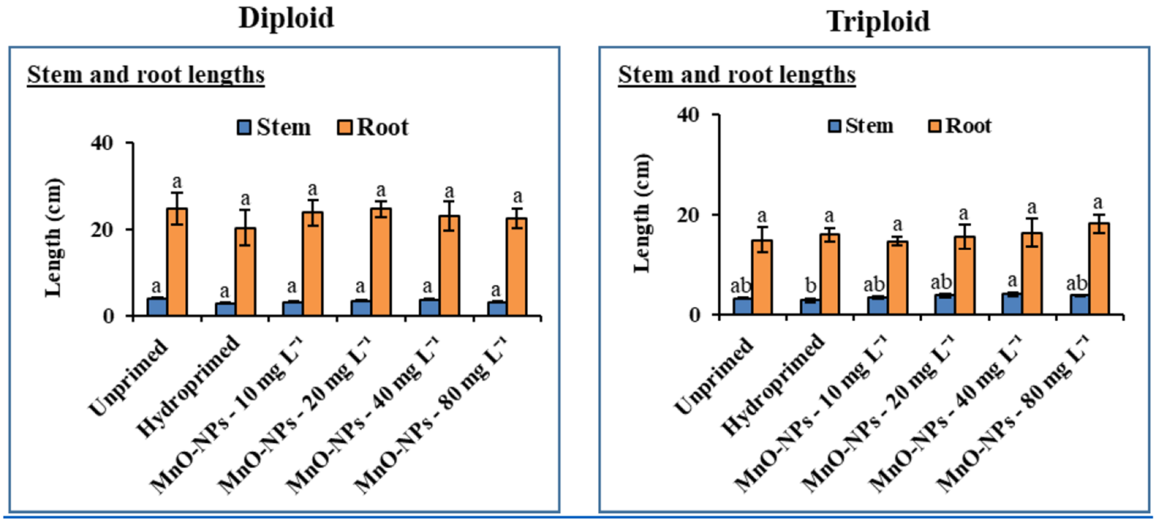

Figure S1. Effect of seeds priming with different concentrations of MnO-NPs on the stem and root lengths of 8-day old diploid and triploid watermelon seedlings. The significant differences ($P < 0.05$) among different are shown by different alphabets, based on a post hoc Tukey test.

© 2021 by the authors. Licensee MDPI, Basel, Switzerland. This article is an open access article distributed under the terms and conditions of the Creative Commons Attribution (CC BY) license (http://creativecommons.org/licenses/by/4.0/).
